# A Review of Microbial Mediated Iron Nanoparticles (IONPs) and Its Biomedical Applications

**DOI:** 10.3390/nano12010130

**Published:** 2021-12-31

**Authors:** Muhammad Nadeem, Rijma Khan, Nausheen Shah, Ishrat Rehman Bangash, Bilal Haider Abbasi, Christophe Hano, Chunzhao Liu, Sana Ullah, Syed Salman Hashmi, Akhtar Nadhman, Jonathan Celli

**Affiliations:** 1 Department of Biotechnology, Institute of Integrative Biosciences, CECOS University, Peshawar 25100, Pakistan; m.nadeem@cecos.edu.pk (M.N.); rijma.khan@cecos.edu.pk (R.K.); nausheen.khan@cecos.edu.pk (N.S.); ishrat.bangash@cecos.edu.pk (I.R.B.); 2 Department of Biotechnology, Quaid-i-Azam University, Islamabad 45320, Pakistan; bhabbasi@qau.edu.pk (B.H.A.); qazisana55@gmail.com (S.U.); sshashmi10@gmail.com (S.S.H.); 3 Laboratoire de Biologie des Ligneux et des Grandes Cultures (LBLGC), Plant Lignans Team, INRAE USC1328, Eure Et Loir Campus, Université d’Orléans, F28000 Chartres, France; hano@univ-orleans.fr; 4 State Key Laboratory of Bio-fibers, Eco-textiles Institute of Biochemical Engineering, College of Materials Science and Engineering, Qingdao University, Qingdao 266071, China; czliu@ipe.ac.cn; 5 State Key Laboratory of Biochemical Engineering, Key Laboratory of Green Process and Engineering, Institute of Process Engineering, Chinese Academy of Sciences, Beijing 100190, China; 6 Department of Physics, University of Massachusetts, Boston, MA 02125, USA; Jonathan.Celli@umb.edu

**Keywords:** green synthesis, nanoparticles, iron oxide, antimicrobial, anticancer

## Abstract

Nanotechnology is a booming avenue in science and has a multitude of applications in health, agriculture, and industry. It exploits materials’ size at nanoscale (1–100 nm) known as nanoparticles (NPs). These nanoscale constituents are made via chemical, physical, and biological methods; however, the biological approach offers multiple benefits over the other counterparts. This method utilizes various biological resources for synthesis (microbes, plants, and others), which act as a reducing and capping agent. Among these sources, microbes provide an excellent platform for synthesis and have been recently exploited in the synthesis of various metallic NPs, in particular iron. Owing to their biocompatible nature, superparamagnetic properties, small size efficient, permeability, and absorption, they have become an integral part of biomedical research. This review focuses on microbial synthesis of iron oxide nanoparticles using various species of bacteria, fungi, and yeast. Possible applications and challenges that need to be addressed have also been discussed in the review; in particular, their antimicrobial and anticancer potentials are discussed in detail along with possible mechanisms. Moreover, some other possible biomedical applications are also highlighted. Although iron oxide nanoparticles have revolutionized biomedical research, issues such as cytotoxicity and biodegradability are still a major bottleneck in the commercialization of these nanoparticle-based products. Addressing these issues should be the topmost priority so that the biomedical industry can reap maximum benefit from iron oxide nanoparticle-based products.

## 1. Introduction

Nanotechnology has revolutionized every field of science and technology and has a multitude of applications [1,2]. In the past, nanotechnology has seen exponential growth with numerous practical applications in health, electronics, cosmetics, and agriculture [3]. In the biomedical field, it has been utilized in diagnostics and treatment of various disorders [4]. The core building blocks of nanotechnology are nanoparticles (NPs). These nanoscale entities range in size from 1–100 nm [5,6]. In contrast to their bulk counterparts, NPs possess unique physiochemical, electrical, magnetic, and thermal properties [7]. Among other metallic NPs, iron NPs (IONPs) have been used extensively in biomedical applications owing to their small size, superparamagnetic properties, and lower biocompatibility. It has also been used in bioprocessing, targeted delivery, imaging, tissue engineering, and disease management [8,9,10]. In particular, the antimicrobial, anti-larvicidal, and antioxidant therapies are the most notable ones [11].

IONPs are mostly produced via physical and chemical methods [12]. However, these approaches are expensive, laborious, and are not safe for any biomedical purposes [12,13,14]. In order to find a viable approach, scientists used a more sophisticated method: green synthesis. This method offers much better alternatives which are more efficient, cost effective, ecofriendly, and safe. This technique utilizes biological resources such as microbial cells, algae, fungi, and plants [15]. It not only reduces the salt, but also aids in improving their stability and morphology, and reducing toxicity [13].

To date, various biological resources have been exploited in the fabrication of IONPs. However, microbial synthesis of IONPs has proven to be an efficient approach compared to others. Microorganisms can efficiently convert iron ions into IONPs using a variety of secondary metabolites and enzymes [16]. The green derived IONPs are safer, ecofriendly, and exhibit excellent biological potential [9]. Green derived IONPs have been used against various disorders including cancer, microbial infections, and antioxidant therapies [17,18]. Moreover, they have also shown excellent catalytic and imaging potentials [9,19]. This review focuses on microbial mediated IONPs using various species of bacteria, fungus, and yeast. Moreover, their biomedical applications have been discussed in detail, especially regarding cancer and antimicrobial therapies. Furthermore, possible directions and limitations are also highlighted. This review will provide a cogent insight for the researchers in nano-biotechnology.

## 2. Bacterial Mediated Synthesis

Bacterial mediated synthesis has emerged as a sustainable approach for the green synthesis of variety of NPs due to its diversity, adaptability to extreme conditions, and ecofriendly nature [20]. Bacteria have the ability to synthesize NPs both intracellularly and extracellularly, depending upon the bacterial strain used [21]. Table 1 provides a list of bacteria with the ability to produce IONPs using intracellular or extracellular mechanisms. A number of researchers have utilized bacteria as nano-factories for IONP synthesis. Magnetic IONPs were synthesized extra-cellularly using *Bacillus cereus strain* HMH1. As a result, highly stable spherical shaped NPs with an average size of 29.3 nm were produced. Bacterial secondary metabolites containing carboxyl groups with primary amines were found to be responsible for IONPs biosynthesis. The formulated polysaccharide coated IONPs mediated by *Staphylococcus warneri* have also been reported [22,23]. The resulting NPs were spherical in shape with an average diameter of 34 nm. The synthesized NPs exhibited high biocompatibility and could be an excellent tool for targeted therapies. Cytoplasmic extract of *Lactobacillus casei* have also been employed for the biosynthesis of spherical IONPs with an average size of 15 nm. [22]. Extracellular biosynthesis of IONPs was reported by Sundaram et al. (2012) using *Bacillus subtilis* extract [24]. The resulting IONPs were spherical shaped with an average size of 60 to 80 nm. The functional groups responsible for the reduction and capping of the said IONPs included Hydroxyl, alkyl, and carboxylic groups that caused the reduction of bulk salt into Fe_2_O_3_ NPs. Rajeswaran et al. (2020) used *Streptomyces* sp. (SRT12) for the synthesis of quasi-spherical IONPs with an average size of 65.0 to 86.7 nm. The resulting NPs showed potent antioxidant and bactericidal activity [17]. *Proteus vulgaris* (ATCC-29905) mediated IONPs also proved to be excellent anticancer and antimicrobial agents [18].

A number of researchers have conducted similar studies which have been summarized in Table 1. Difference in synthesis factors (pH, temperature, and species difference) significantly affects the characteristics (size and shape) of IONPs. If the synthesis route is accurately sustained and elucidated, it will improve the synthesis yield, and better morphologies and sizes will be obtained which could be scaled for commercial scale.

## 3. Fungus Mediated Synthesis

Similar to bacteria, myogenic synthesis has also gained a lot of attention due to its biocompatibility, low toxicity, comparatively economic nature, effortless synthesis, and eco-friendly protocols. Mycogenic synthesis of IONPs may be either extracellular or intracellular (Table 2), depending upon the type of microbial species used [44]. *Aspergillus flavus* has been used for the extracellular synthesis of spherical IONPs with an average size of 28–33 nm. Different functional groups such as alkyl, carboxylic acid, hydroxyl, and amide were responsible for the reduction and capping of *Aspergillus flavus* mediated IONPs [45]. Baskar et al. (2017) synthesized IONPs ranging in size from 40–100 nm using *Aspergillus terreus.* The resulting NPs showed remarkable anti-cancer potency, suggesting that IONPs can be employed in the future as a potential anticancer drug [46]. *Trichoderma asperellum*, *Phialemoniopsis ocularis*, and *Fusarium incarnatum* have also been reported for the biogenic synthesis of IONPs [47]. *Aspergillus niger* has been reported for the synthesis of magnetite IONPs. Synthesized NPs were characterized using XRD and SEM, which revealed the production of spherical shaped IONPs with average size of 15 to 18 nm. The biogenic IONPs showed excellent hyperthermia phenomena in cancer [44]. Adeleye et al. (2020) reported the use of *Rhizopus stolonifer* for the synthesis of IONPs. The NPs were stabilized by secondary metabolites containing a variety of functional groups such as thiol, carboxylic acid, hydroxyl, and alkyl groups [48]. Endophytic fungi *Penicillium oxalicum* has also been used for the synthesis of spherical IONPs with an ability to effectively catalyze degradation of methylene blue dye [19]. A detailed account of myogenic IONPs, their characterization, and potential applications has been provided in Table 2.

From previous studies, it has been shown that fungus could be an excellent candidate for synthesis of IONPs as compared to other biological sources. It has better yield, more complex proteins, and metabolites which can reduce and stabilize metal salts for longer periods of time. However, more detailed studies are needed to decipher the synthesis process in detail and reaction parameters should also be evaluated to achieve better yield and stability.

In addition, yeast is among some of the valuable species for the mass production of different kinds of nanoparticles. *Saccharomyces cerevisiae* and *Cryptococcus humicola* have been reported for the synthesis of magneto-sensitive IONPs. For the synthesis, the aforementioned species were incubated on laboratory temperature (22–25 °C) followed by the addition of precursor salt. The resulting mixture was then observed under magnetic field to check for the formulation of IONPs [49]. *Candida bombicola* has also been used for the synthesis of sophorolipids-functionalized IONPs. The synthesized NPs were characterized using TEM, FTIR, and XRD. The TEM results revealed crystalline IONPs with an average size of 8.5 nm and 4.5 nm. FTIR results indicated the presence of a carboxylic functional group [50]. Though very little has been revealed regarding the biosynthesis of IONPs from yeast to date, considering their rich metabolomic and proteomic profile, further studies should be directed to evaluate their potential and biosynthesis mechanism. Many other studies have also been conducted on the biogenic IONPs, as shown in Table 2.

**Table 2:** Fungus/yeast mediated iron nanoparticles.

S.no	Species	Location of Synthesis	Characterization	Functional Group	Shape	Size (nm)	Ref.
1	*Alternaria alternata*	Extracellular	SEM, TEM, and EDX	NR	Cubic shape	3–9	[4]
2	*Pochonia chlamydosporium*	Both Extracellular and Intracellular	TEM and FTIR	NR	NR	20–40	[10]
	*Aspergillus fumigatus*	Both Extracellular and Intracellular	TEM and FTR	NR	NR	20–40	[10]
3	*Fusarium Oxysporum*	Extracellular	TEM and FTIR	NR	Spherical	20–40	[44]
	*Actinomycetes specie*	Extracellular	TEM and FTR	NR	Spherical	20–40	[44]
4	*Aspergillus oryzae*	NR	TEM and FTIR	NR	----	10 and 24.6	[51]
5	*Pochonia chlamydosporium*	Intracellular	TEM and FTR	NR	Spherical	4–80	[10]
6	*Pleurotus specie*	Intracellular	TEM and FTIR	OH, NH_2_, and COOH	NR	----	[52]
7	*Fusarium oxysporum*	Extracellular	TEM and FTR	Amide I and II	Cube	10–40	[53]
	*Verticillium specie*	Extracellular	TEM and FTIR	Amide I and II	Cube	10–40	[53]
10	*Aspergillus specie*	Extracellular	TEM, Atomic Absorption Spectrophotometry	NR	NR	50–20	[19]
11	*Aspergillus japonicus*	Extracellular	XRD, SEM, and EDS	NR	Cubic	60–70	[54]
12	*Neurospora crassa*	NR	SEM, XRD, EDX, and FTIR	OH, C–H, and Fe–O	Coralline appearance,	50	[55]
13	*Trichoderma specie*		UV-Vis and FTIR	C–H, C=O, C≡N, C=H, and OH	NR	----	[56]
	Yeast						
14	*Cryptococcus humicola*	NR	TEM and X-rays	NR	Spherical	8–9	[49]
15	*Candida bombicola*	Extracellular	TEM, FTIR, and XRD	COOH		8.5–4.5	[50]

## 4. Antimicrobial Potential of IONPs

Over the last few years, the emergence of microbial infections has increased dramatically. The rise of multidrug-resistant bacteria (MDR) is further worsening the situation and has become a global health challenge [6]. Recently, nanotechnology-based therapies have been exploited in disease diagnostics and formulations of novel therapeutic drugs against numerous diseases [3,57,58]. Among other NPs, green synthesized IONPs have also been exploited against various pathogenic strains of bacteria [4]. Due to their biocompatibility, safety, and ecofriendly nature, these nanoscale materials have attracted great interest as a novel antimicrobial agent and have been tested against a wide range of infectious pathogens [5,7,59] (Table 3). The antimicrobial potential of these NPs have not been clearly depicted; however, it is suggested that they kill microbes in the same way as their chemical counters [8]. The added advantage in the case of biosynthesized NPs, however, is the addition of capping agents. As most of the capping agents themselves possess antimicrobial potency, the ultimate antimicrobial potential of resulting NPs can be improved. Mostly, NPs kill microbial cells via diverse mechanisms including membrane destruction, organelles damage, biomolecular distortion, and by interfering with nucleic acid or protein biosynthesis in bacterial cells [3,9,57,58].

**Figure 1:**
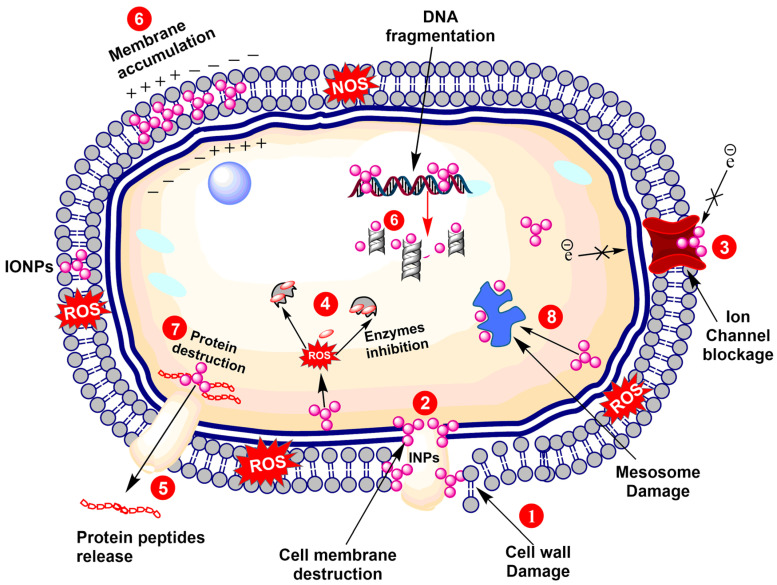
Antibacterial potential of iron nanoparticles (INPs). (1) Cell wall destruction via interfering the normal homeostasis; (2) Cell membrane damage is caused by disorientation of the lipid bilayer via ROS production; (3) Ion channel misconfiguration occurs when transporter proteins are damaged; (4) Enzyme physiology is disrupted via inhibition of their catalytic domains; (5) Nucleic acid is damaged leading to fragmentation of DNA and RNA; (6) Biomolecules disruption occurs, in particular, in proteins and NPs; (7) Proteins denaturation via ROS; and (8) Organelles damage, in particular, mesomes.

Bacterial cells are mostly killed via production of superoxide radicals (O^2−^), hydroxyl radicals (−OH), hydrogen peroxide (H_2_O_2_), and singlet oxygen (O_2_), collectively known as reactive oxygen species (ROS). ROS cause severe damage to nucleic acids and proteins in the microbial cell [10]. NPs interact with membrane proteins (thiol groups) and cause oxidative stress which results in protein denaturation and membrane impermeability. All of this eventually leads to microbial death [5]. Besides membrane disruption, it can also distort structural integrity and cellular architecture [8]. The antibacterial potential of IONPs is elucidated in Figure 1. The biogenic IONPs have also shown great potential to kill both Gram-negative and Gram-positive bacteria, but due to the complex structure of Gram-negative bacteria, it is more effective against Gram-positive bacterial strains [9,11].

These nanoantibiotics have a wide range of advantages over the traditional ones, such as they are less susceptible to microbial resistance; they may be functionalized to numerous preferred target sites; and the possibility of stimulating them with other sources such as pH, heat, light, and magnetic field [6,59]. The biogenic NPs have also shown remarkable antimicrobial potential against a wide range of microbial species and can combat over the rising threat of MDR [59]. In particular, when used along with other conjugates, they inhibited the biofilm formation and showed potent biocidal potential [14]. Despite the growing knowledge on antimicrobial activity against MDR and their strong antimicrobial potential, more studies are required to address their toxicity and elucidate their antimicrobial mechanism in in vivo models. Furthermore, in order to achieve optimal antimicrobial activity, the synthesis process should be optimized to avoid the size and morphological variability.

## 5. Anticancer Activity

**Figure 2:**
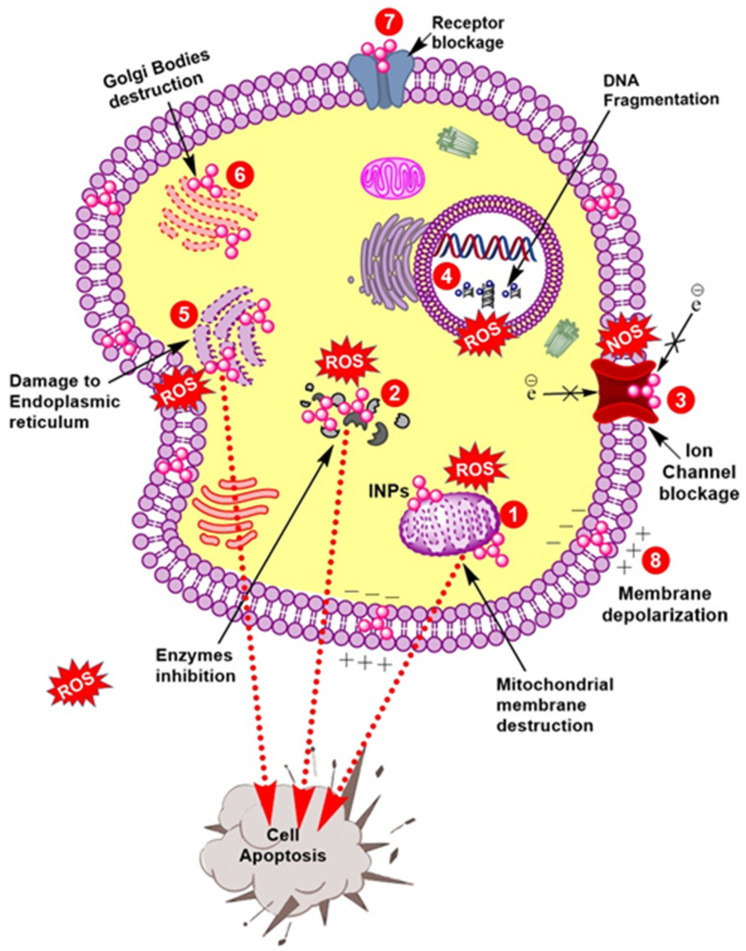
Anticancer potential of microbes mediated Nanoparticles. (1), (2), (5), and (6) Iron nanoparticles interfere with organelles and enzymes functioning, particularly in mitochondria, endoplasmic reticulum, and Golgi bodies via reactive oxygen species (ROS) production and induces apoptosis. (3) Ion channel blockage leads to death of cancerous cells. (4) INPs kills cancerous cells by breaking nucleic acids, particularly in DNA. (8) Membrane polarity is disturbed.

Cancer is the second leading cause of deaths after cardiovascular diseases [6]. To date, no proper treatment is available for cancer; however, the quest to find novel anticancer agents is continuous [57]. Recently, nano-frontier has been exploited in various disease management. Among other NPs, iron has been exploited the most in diagnostics, treatment, or formulation of cancer drugs [58]. These therapeutic properties are attributed to their strong stability, biocompatibility, and specificity against diverse cancer cells [8,31,59]. Additionally, harnessing their magnetic hyperthermia potential can be used to kill cancerous cells selectively [11]. In the past, IONPs have been used in treatment of various cancers such as breast cancer, glioblastoma cancer, liver cancer (Hepatoma H22 cells), leukemia promyelocytic (HL60 cells), cellosaurus cell line (MOLT-4 cell), and prostate cancer [17]. In all treatments, IONPs exhibited strong cytotoxic potential against the aforementioned cancer cell lines. Microbe-mediated IONPs escalate oxidative stress and kill the cells by impeding their cell division and distorting macromolecules framework which ultimately leads to cell death via activating apoptosis [17,18,22,23,46]. The anticancer potential of IONPs is depicted in Figure 2. When mixed with other anticancer drugs, it significantly accelerated the antitumor potential [3]. Considering their anticancer potential, these nanoparticles can be tested in in vivo models to determine their effectiveness; however, their toxicity must be taken into account when it comes to humans.

## 6. Other Potential Applications

Beside the antimicrobial and anticancer potential of IONPs, they have also been exploited in drug delivery, antioxidant therapies, and catalysis [17,27]. For instance, they have been used in the degradation of methyl violet, chlorinated pollutants, and methylene blue dyes [35,48]. However, the current knowledge regarding their catalytic mechanism is miniscule, which needs to be addressed in order to employ them as a catalytic agent in remediation process. In agriculture practices, microbial mediated IONPs have been used on a test basis and have shown promising results as compared to chemical peers [28]. With such tremendous potential, they are believed to have a promising future in farming and could be used in the fabrication of novel fertilizers, bio-control agents, and advanced sensing technologies. However, certain limitations (Cytotoxicity and Eutrophication) need to be addressed before translating this technology into fields.

## 7. Conclusions

Considering the biocompatibility, safety, and minimal toxicity of green synthesized IONPs, they have been exploited in diagnosis, management, and treatment of various diseases. The most notable application in the medical field is their antimicrobial potential, which is attributed to their smaller size, large surface area, and biocidal potential. To date, a clear picture of the antimicrobial mechanism of action has not been elucidated. The antimicrobial mechanism of green synthesized IONPs is believed to be associated with reactive oxygen species (ROS) production, which can interfere with normal cellular metabolism and hemostasis across bacterial walls, shutting down organelles’ membranes and destroying membranes and nuclear materials. Moreover, green synthesized IONPs have also shown significant antimicrobial action against MDRs, which promises to provide leverage against antimicrobial resistance in the near future. With the currently limited literature, further studies are required to evaluate their in vivo efficacy and elucidate their antimicrobial potential in detail. Green synthesized IONPs have also shown excellent anti-cancer potential in many in vitro based studies. Green synthesized IONPs have a unique ability to induce apoptosis in cancer cells selectively via destruction of membranes, fragmenting the nuclear materials or hampering the enzyme and organelles functioning. However, little has been explored regarding their anticancer potential which needs to be studied in detail in both in vitro and in vivo experiments. Green synthesized IONPs have also been used in diagnostics and treatment of other diseases, but very little is known. Nevertheless, they are likely to have a dazzling future in the management of other incurable diseases, including hypertension and diabetes. Beside their medical applications, green synthesized IONPs have also been used in various agricultural practices and could be used as alternative to bio fertilizers and bio-control agents. With such a multitude of applications and promising results in various fields of science, the only hurdle in its commercialization is its toxicity. For now, toxicity of NPs remains a major bottleneck in translating these materials from lab to industry, which needs to be addressed further.

## Figures and Tables

**Table 1:** Bacterial mediated Iron nanoparticles.

S.no	Species	Location of Synthesis	Characterization	Functional Group Involved in Reduction	Shape	Size (nm)	Ref
1	*Actinobacter* sp.	Extracellular	TEM, XRD, and FTIR	Fe–O bond	Crystal	50	[25]
2	*Shewanella oneidensis*	NR	TEM, XRD, and AFM	NR	Pseudo-hexagonal shape	11, 30, 99	[11]
3	*Magnetospirillum gryphiswaldense*	Extracellular	DLS, TEM, SAXS, and FTIR	NR	Polydispersed	25–55	[26]
4	*Magnetotactic bacteria*	Intracellular	TEM	NR	Spherical	25–50	[27]
5	*Paenibacillus polymyxa*	NR	TEM, FTIR, and UV-Vis	O–H, C–H, CO_2_NH_3_, C=O, C=C, and N–H	Spherical	26.65	[28]
6	*Geobacter* *sulfurreducens*	Extracellular	PXRD and TEM	NR	NR	10–50	[29]
7	*Klebsiella Oxytoca*	NR	----	NR	NR	2–5	[30]
8	*Lactobacillus Fermentum*	Intracellular	XRD and TEM	NR	Spherical	10–15	[31]
9	*Gluconacetobacter xylinus*	Intracellular	SEM	NR	NR	50	[16]
10	*Proteus mirabilis*	NR	XRD, EDX, TEM, UV-Vis, and Zeta sizer	NR	Spherical	1.44–1.92	[32]
11	*Escherichia coli*	Extracellular	FESEM, EDX, TEM, and UV-Vis	NR	Spherical	23	[33]
	*Pseudomonas aeruginosa*	Extracellular	FESEM, EDX, TEM, and UV-Vis	NR	Spherical	23	[33]
12	*Desulfotomacculum acetoxidans*	NR	SEM-EDS and XRD	NR	NR	21	[34]
13	*Pseudomonas stutzeri*	NR	XRD, FTIRUV-Vis, SEM, and TEM	O–H, C–H, Fe–O, C=C, and N–H	NR	10–20	[35]
14	*Desulfovibrio*	NR	TEM, XRD, and FTIR	NR	NR	19	[34]
15	*Bacillus subtilis*	Extracellular	FE-SEM, TEM, XRD, FTIR, DLS, and VSM	O–H, C–H, Fe–O, C=C, and N–H	Rhombohedral	37–97	[36]
	*Bacillus pasteurii*	NR	FE-SEM, TEM, XRD, FTIR, DLS, and VSM	O–H, C–H, Fe–O, C=C, N–H	Rhombohedral	37–97	[36]
	*Bacillus licheniformis*	NR	FE-SEM, TEM, XRD, FTIR, DLS, and VSM	O–H, C–H, Fe–O, C=C, and N–H	Rhombohedral	37–97	[36]
16	*Leptothrix ochracea*	Extracellular	SEM, EDX, and XRD	NR	hollow tube	100	[37]
17	*Caulobacter crescentus*	NR	FE-SEM, XRD, AFM, and EDAX	NR	Spherical	50	[38]
18	*Geobacter* *specie*	NR	XRD, SEM-EDX, TEM-EDX, and ICP-AES	NR	NR	50–60	[39]
19	*Streptococcus suis*	NR	EXAFS and XRD	NR	NR	----	[40]
20	*Magnetospirillum gryphiswaldense*	Extracellular	TEM	NR	NR	----	[41]
21	*Thiobacillus thioparus*	NR	SDS PAGE Gel	NR	NR	----	[42]
22	*Alcaligenes faecalis*	Extracellular	SEM, EDX, and FTIR	HO–NH_3_	NR		[43]

**Table 3:** Microbial species tested against various microbes mediated **IONP**.

S.no	Species	Inhibition Method	Activity Against	Ref.
1	*Proteus vulgaris*	Disc Diffusion method	*Salmonella enterica*, *Escherichia coli*, *Vibrio cholera*, *Salmonella typhi*, and *Staphylococcus epidermidis*	[18]
2	*Streptomyces* (SRT12)	Disc Diffusion method	*Bacillus subtilis*, *Staphylococcus aureus*, *Klebsiella pneumoniae*, *Shigella flexneri*, and *Escherichia coli*.	[17]
3	*Proteus mirabilis*	Well-diffusion method	*E. coli*, *Salmonella typhi*, *P. aeruginosa*, *Clostridium perfringens*, *Aspergillus Brasiliensis*, and *Candida Albicans*	[32]
4	*Alternaria alternata*	Well-diffusion method	*Bacillus subtilis*	[4]
5	*Fusarium oxysporum*	Disc diffusion method	*Staphylococcusaureus*, *Klebsiella pneumoniae*, *Proteus vulgaris*, *Pseudomonas aeruginosa*, and *Escherichia coli*	[24]
6	*Aspergillus flavus*	Diffusion agar technique	*Staphylococcus aureus*, *Escherichia coli*, *Candida albicans*, and *Aspergillus Fumigatus*	[45]
7	*NPs-penicillin G conjugates*	Disc Diffusion method	*Staphylococcus aureus*	[60]

## Data Availability

Not applicable.
